# Association Between Body Mass Index and Breast Cancer Incidence in Non-Hispanic White and Black Women: Methodological Considerations From a Secondary Analysis of the Vitamin D and Omega-3 Trial Cohort

**DOI:** 10.7759/cureus.107664

**Published:** 2026-04-24

**Authors:** Oluwatosin E Oluwole, David R Jacobs, Taiwo O Aremu

**Affiliations:** 1 Division of Epidemiology and Community Health, School of Public Health, University of Minnesota, Minneapolis, USA; 2 Division of Environmental Health Sciences, School of Public Health, University of Minnesota, Minneapolis, USA; 3 Department of Pediatrics, School of Medicine, University of Minnesota, Minneapolis, USA; 4 College of Pharmacy, Touro University California, Vallejo, USA

**Keywords:** black women, body mass index (bmi), breast cancer incidence, cohort study, non-hispanic white women, obesity, older women, racial and ethnic disparities

## Abstract

Introduction: Obesity and age are established risk factors for breast cancer, but racial differences in the association between body mass index (BMI) and breast cancer incidence remain incompletely understood. This study examined the association between BMI and breast cancer incidence among non-Hispanic White and Black women in the VITamin D and OmegA-3 TriaL (VITAL) cohort and secondarily considered methodological issues that may arise when clinical trial data are analyzed observationally.

Methods: We conducted a secondary observational analysis of women enrolled in the VITAL cohort, treating trial participants as a cohort rather than comparing randomized treatment arms. After excluding men and women of races/ethnicities other than non-Hispanic White or Black, 11,377 women aged 55-90 years at baseline with no previous history of breast cancer were included. Using SAS OnDemand software (SAS Institute, Cary, NC), we examined the association between BMI and the incidence of breast cancer with Cox proportional hazards regression models.

Results: The study sample of 218 women with breast cancer included 84% non-Hispanic White women (n = 183) and 16% Black women (n = 35). Age and White race were each positively related to incident breast cancer in unadjusted models (both p < 0.001). There was a positive nonlinear relationship between BMI and breast cancer (p = 0.0072, df = 1, x^2^ = 7.23). On more detailed examination, the increasing breast cancer risk was found mainly across lower levels of BMI and younger ages in these non-Hispanic White and Black women.

Conclusion: Breast cancer incidence showed a nonlinear association with BMI and age, increasing across lower ranges but attenuating at higher BMI levels and older ages. Because this was a secondary analysis of a selected trial cohort, the unexpected attenuation should be interpreted cautiously rather than as evidence of a physiologic plateau.

## Introduction

Breast cancer is the most commonly diagnosed cancer among women in the United States (US), excluding skin cancers, and remains the second leading cause of cancer deaths among women after lung cancer [1-3]. Contemporary US estimates indicate that approximately 321,910 new invasive breast cancers and 42,140 breast cancer deaths are expected among women in 2026, and incidence rates have increased by about 1% per year in recent years [3]. Among known risk factors for breast cancer are female sex, increasing age, family history and genetic susceptibility, reproductive and hormonal factors, and obesity [4].

Body mass index (BMI), calculated as an individual's weight in kilograms divided by their height in meters squared, is a standard metric for body fatness in humans [5]. Four BMI categories are commonly used in adults: underweight (<18.5 kg/m²), normal weight (18.5 to <25.0 kg/m²), overweight (25.0 to <30.0 kg/m²), and obesity (≥30.0 kg/m²) [6]. Although BMI is only one component of breast cancer risk, several biologic mechanisms have been proposed to explain its association with postmenopausal breast cancer. Greater adiposity may increase estrogen production and leptin signaling, and obesity is also associated with lower sex hormone-binding globulin (SHBG) levels and insulin resistance, all of which may influence breast cancer risk [7-9].

Although recent cohort and meta-analytic studies have shown a generally positive association between increasing BMI and postmenopausal breast cancer incidence [10,11], whether this association differs between non-Hispanic White and Black women in the United States has not been well-explored. The primary epidemiologic objective of this study was to examine the association between BMI and breast cancer incidence among non-Hispanic White and Black women in the VITamin D and OmegA-3 TriaL (VITAL) cohort, with additional consideration of age and race [12]. We hypothesized that the association between higher BMI and breast cancer incidence would be stronger among Black women than among non-Hispanic White women. Because this analysis used observational data from a selected randomized trial population rather than comparing randomized treatment arms, a secondary methodological objective was to assess how features of the trial-cohort data may influence the interpretation of unexpected or nonlinear exposure-disease patterns.

This article was previously presented as a poster at the 2023 University of Minnesota School of Public Health Research Day on April 4, 2023.

## Materials and methods

Study design

The VITAL trial was a nationwide, randomized, double-blind, placebo-controlled 2 x 2 factorial trial designed to test whether vitamin D3 and marine omega-3 fatty acid supplementation reduced the risk of cancer and cardiovascular disease among adults without these conditions at baseline [12,13]. Randomization occurred from November 2011 to March 2014, randomized treatment ended on December 31, 2017, and annual follow-up continued thereafter [13]. The parent trial was conducted primarily through postal and electronic communication, with a Boston-area clinic subcohort undergoing more detailed in-person assessments [13]. The present study used VITAL observationally as a cohort of women rather than as a comparison of randomized treatment groups. Data were obtained through the Project Data Sphere access portal as a public-use release.

Study population

The VITAL study included 25,871 US adults. Eligible participants were men aged 50 years or older and women aged 55 years or older with no history of cancer (except nonmelanoma skin cancer), myocardial infarction, stroke, transient ischemic attack, or coronary revascularization, and without the major safety exclusions described in the VITAL design paper [12,13]. African Americans were oversampled by design, and participants were enrolled nationwide [12,13].

**Figure 1:**
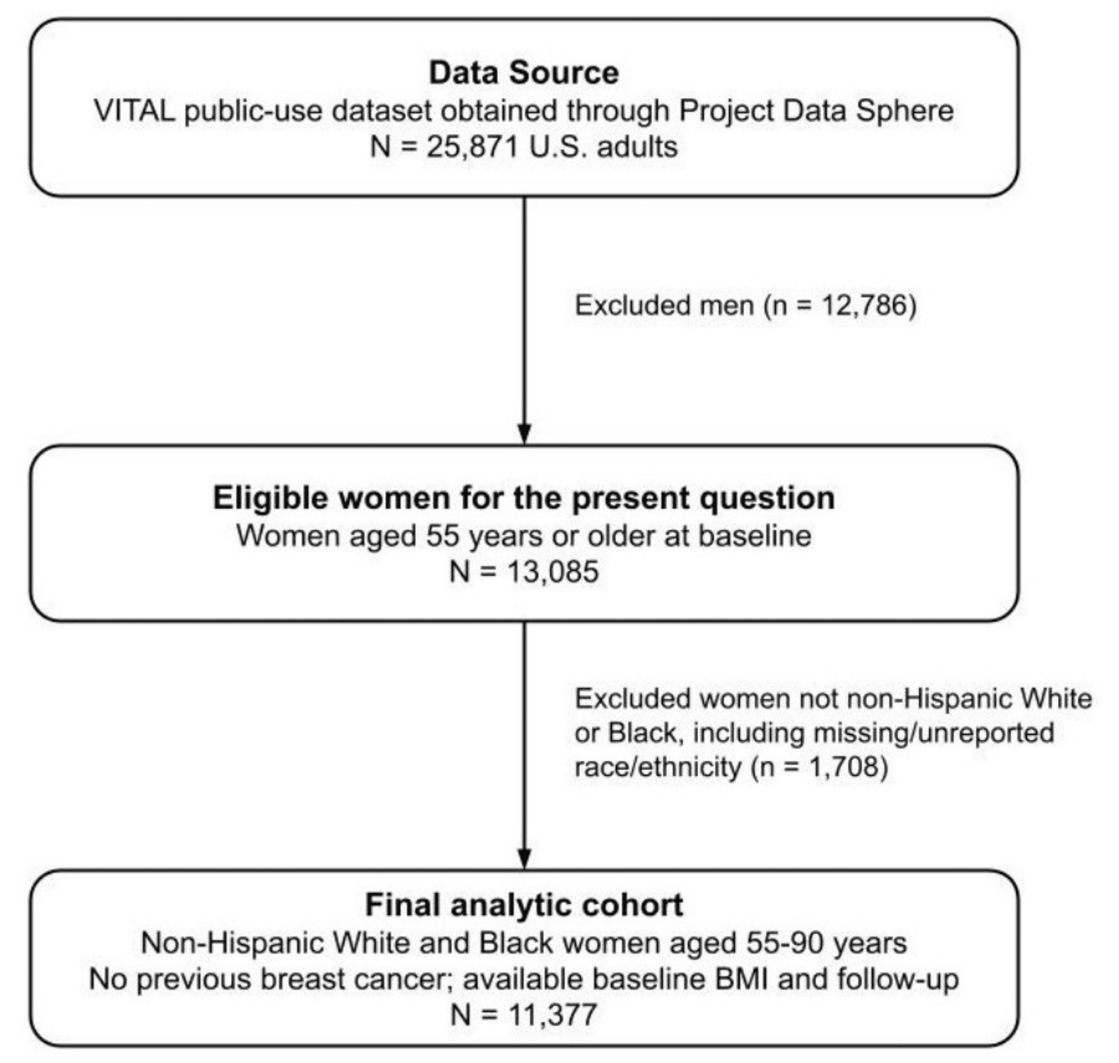
Flow diagram of participant selection for the analytic cohort.

The analytic sample consisted of women aged 55-90 years at baseline with no previous history of breast cancer. We included women in this age range because VITAL enrolled women beginning at age 55, which is relevant to postmenopausal breast cancer risk and reflects the age range available in the parent trial. Men were excluded because the study question focused on breast cancer incidence among women (n = 12,786 excluded). We then restricted the sample to non-Hispanic White and Black women because these groups had sufficient numbers for a targeted comparison within VITAL and because evidence comparing BMI and breast cancer associations across these two groups remains limited. Participants from other racial/ethnic groups, including Asian, Hispanic, Native American, other, and missing or unreported race/ethnicity, were excluded because their smaller and heterogeneous counts would not support stable group-specific interpretation (n = 1,708 excluded). The final analytic sample included 11,377 women. The derivation of the final analytic cohort is shown in Figure 1.

Exposure

Height and body weight were self-reported on the baseline questionnaire, and BMI was calculated as weight (kg) divided by height (m^2^). In-person anthropometric measurements were available only in a Boston-area clinic subcohort, but the present analysis relied on the questionnaire-based public-use data [13].

Outcome variable

The primary outcome was incident breast cancer during follow-up through the public-use dataset. Participants completed annual follow-up questionnaires regarding endpoint occurrence; reported breast cancers were adjudicated by blinded study physicians through medical record review with final confirmation based on histology or cytology data; and deaths were ascertained through the National Death Index-Plus and other sources [13]. In the parent trial, yearly questionnaire response rates averaged 93%, and mortality follow-up exceeded 98% [13].

Covariates

Age, race, diabetes status, and current smoking status were included as covariates in the adjusted models. Age was included because it is a strong determinant of breast cancer incidence. Race was included because a comparison between non-Hispanic White and Black women was central to the study question. Diabetes and current smoking were selected a priori because they are related to BMI or general cancer risk and were available in the public-use dataset. Thus, the adjustment set was based on existing epidemiologic relevance, the study question, and data availability. Other potentially relevant variables, including hormone therapy, reproductive history, family history, screening history, diet, physical activity, and socioeconomic measures, were not available in the public-use database.

Statistical analysis

Descriptive analyses were conducted to summarize baseline characteristics of non-Hispanic White and Black women. Continuous variables are presented as means and standard deviations, and categorical variables are presented as frequencies and percentages.

BMI was categorized according to the WHO adult BMI standards as underweight (<18.5 kg/m²), normal weight (18.5 to <25.0 kg/m²), overweight (25.0 to <30.0 kg/m²), and obese (≥30.0 kg/m²). Normal weight was used as the reference category.

Cox proportional hazards regression models were used to examine the association between BMI category and breast cancer incidence. We first fit an unadjusted model and then fit a multivariable model adjusted for age, race, diabetes status, and current smoking status. Age was included because it is a strong determinant of breast cancer incidence. Race was included because a comparison between non-Hispanic White and Black women was central to the study question. Diabetes and current smoking were selected a priori because they are related to BMI or general cancer risk and were available in the public-use dataset. Non-Hispanic White women were used as the race reference group; therefore, the race estimate compares Black women with non-Hispanic White women. Diabetes and current smoking were modeled as yes/no variables.

To evaluate whether the associations of BMI and age with breast cancer incidence were nonlinear, additional Cox models included quadratic terms for BMI and age. Participants who did not develop breast cancer during available follow-up were right-censored at their last available follow-up time or at the end of available follow-up. Regression coefficients were exponentiated to obtain hazard ratios (HRs), and 95% confidence intervals (CIs) were calculated from the corresponding model estimates. All statistical tests were two-tailed, with the significance level set at 0.05. Analyses were performed using SAS OnDemand for Academics (SAS Institute, Cary, NC).

Ethical consideration

This study was conducted in accordance with the Declaration of Helsinki and approved by the Institutional Review Board of the University of Minnesota as a human research exempt study (IRB ID: STUDY00018191; date of approval: January 27, 2023).

## Results

After excluding men and women from race/ethnic groups other than non-Hispanic White and Black, the final sample included 11,377 women, of whom 2,984 (26.2%) were Black and 8,393 (73.8%) were non-Hispanic White. Available baseline characteristics, including age in quartiles, BMI, smoking status, and diabetes status, are shown in Table 1.

**Table 1:** Baseline characteristics of the participants.

Variables		Black women (N = 2,984); n (row %)	Non-Hispanic White women (N = 8,393); n (row %)	Total participants for each category (N = 11,377); n (column %)
Age group (years) (Range = 55-90 years)	Quartile 1: 55-62	1,596 (57.9)	1,163 (42.2)	2,759 (24.3)
	Quartile 2: 63-67	663 (20.6)	2,563 (79.5)	3,226 (28.4)
	Quartile 3: 68-71	373 (14.2)	2,259 (85.8)	2,632 (23.1)
	Quartile 4: 72-90	352 (12.8)	2,408 (87.3)	2,760 (24.3)
Baseline body mass index (kg/m^2^)	Underweight (<18.5)	15 (9.5)	143 (90.5)	158 (1.4)
	Normal (18.5-24.9)	467 (12.5)	3,256 (87.5)	3,723 (32.7)
	Overweight (25.0-29.9)	940 (25.1)	2,812 (74.9)	3,752 (33.0)
	Obese (≥30.0)	1,562 (41.7)	2,182 (58.3)	3,744 (32.9)
Diabetes status	Yes	750 (48.9)	783 (51.8)	1,533 (13.5)
	No	2,234 (22.7)	7,610 (77.3)	9,844 (86.5)
Current smoking status	Yes	368 (46.4)	425 (53.6)	793 (7.0)
	No	2,616 (24.7)	7,968 (75.3)	10,584 (93.0)

Among Black female participants, the most frequent age group was 55-62 years. The mean age among Black women was 63.7 years (standard deviation (SD) = 6.4), whereas among non-Hispanic White women, the mean age was 68.8 years (SD = 6.4). The mean BMI was 31.6 kg/m^2^ (SD = 7.2) among Black women and 27.3 kg/m^2^ (SD = 5.9) among non-Hispanic White women. Black women in this analytic subset were younger on average and had higher mean BMI than non-Hispanic White women.

In the time-to-event analysis, 11,159 women (98.1%) did not develop breast cancer during available follow-up and were censored at their last observed follow-up time or at the end of the public-use follow-up period; 218 women (1.9%) developed breast cancer. Among the 218 women who developed breast cancer, 183 (83.9%) were non-Hispanic White and 35 (16.1%) were Black (Table 2).

**Table 2:** Crude incidence of breast cancer by race, age, BMI, diabetes status, and smoking status.

Variables		Breast cancer, N (%)	No breast cancer, N (%)	Total participants for each category
Race	Black women	35 (1.2)	2,949 (98.8)	2,984
	Non-Hispanic White women	183 (2.2)	8,210 (97.8)	8,393
BMI	Underweight	4 (2.5)	154 (97.5)	158
	Normal weight	72 (1.9)	3,651 (98.1)	3,723
	Overweight	79 (2.1)	3,673 (98.3)	3,752
	Obese	63 (1.7)	3,681 (98.3)	3,744
Age	Quartile 1: 55-62	27 (1.0)	2,732 (99.0)	2,759
	Quartile 2: 63-67	57 (1.8)	3,169 (98.2)	3,226
	Quartile 3: 68-71	66 (2.5)	2,566 (97.5)	2,632
	Quartile 4: 72-90	68 (2.5)	2,692 (97.5)	2,760
Diabetes status	Yes	25 (1.6)	1,508 (98.4)	1,533
	No	193 (2.0)	9,651 (98.0)	9,844
Current smoking status	Yes	14 (1.8)	779 (98.2)	793
	No	204 (1.9)	10,380 (98.0)	10,584

Women aged 68-90 years had higher crude breast cancer incidence than women aged 55-67 years; however, incidence did not increase further between the 68-71 and 72-90 years age groups. Crude cumulative incidence by BMI category within each racial group is shown in Table 3. Within non-Hispanic White women, cumulative incidence was 2.80% in underweight women, 2.05% in normal-weight women, 2.42% in overweight women, and 2.02% in obese women. Within Black women, the corresponding incidences were 0.00%, 1.07%, 1.17%, and 1.22%, respectively. Because the underweight group was small (n = 158 overall), estimates for this group should be interpreted cautiously.

**Table 3:** Crude cumulative incidence of breast cancer by race an BMI category.

	Breast cancer, N (%)	No breast cancer, N (%)	Total
Black women			
Underweight	0 (0.00)	15 (100.00)	15
Normal weight	5 (1.07)	462 (98.93)	467
Overweight	11 (1.17)	929 (98.83)	940
Obese	19 (1.22)	1,543 (98.78)	1,562
Non-Hispanic White women			
Underweight	4 (2.80)	139 (97.20)	143
Normal weight	67 (2.05)	3,189 (97.94)	3,256
Overweight	68 (2.42)	2,744 (97.58)	2,812
Obese	44 (2.02)	2,138 (97.98)	2,182

In crude analyses, non-Hispanic White women had higher breast cancer incidence than Black women. In Cox models, the hazard ratio for Black versus non-Hispanic White race was 0.56 (95% CI: 0.38-0.80) before adjustment and 0.69 (95% CI: 0.47-1.03) after adjustment for BMI category, age quartile, diabetes, and current smoking. To evaluate curvature, we fitted proportional hazards models with quadratic BMI and quadratic age terms plus covariates. In age- and race-adjusted continuous models, the BMI squared term was statistically significant (p = 0.0079), and the age squared term was also statistically significant (p = 0.0020), supporting a nonlinear pattern. For the categorical BMI analysis, Table 4 presents the unadjusted model and the fully adjusted model. The category-based hazard ratios were close to 1.0 and did not suggest a strictly monotonic increase across BMI categories.

**Table 4:** Cox proportional hazard models for breast cancer incidence according to BMI categories. Normal weight was the reference category for BMI. Model 1 is unadjusted. Model 2 is adjusted for age, race, diabetes status, and current smoking status. Non-Hispanic White women were the reference group for race; therefore, the race estimate compares Black women with non-Hispanic White women. Diabetes and current smoking were coded as yes/no variables. HR: hazard ratio; CI: confidence interval.

Models and adjustments	Underweight (<18.5 kg/m^2^); HR (95% CI)	Normal weight (18.5 to <25.0 kg/m^2^); HR (95% CI)	Overweight (25.0 to <30.0 kg/m^2^); HR (95% CI)	Obese (≥30.0 kg/m^2^); HR (95% CI)
Model 1: Unadjusted (crude)	1.37 (0.50-3.75)	Ref.	1.10 (0.80-1.51)	0.88 (0.63-1.24)
Model 2: Fully adjusted	1.28 (0.47-3.52)	Ref.	1.18 (0.85-1.62)	1.08 (0.76-1.54)

## Discussion

The findings in this cohort of women in the VITAL trial study suggest that the associations of breast cancer incidence with BMI and age were not strictly linear. Risk appeared to increase across lower BMI levels and younger ages, but the gradient flattened at the highest BMI levels and older ages. The categorized models in Table 4 were directionally consistent with the crude tables but remained imprecise, which is not unexpected given the modest number of breast cancer cases and the lower efficiency of categorized analyses compared with continuous curved models. We therefore interpret the observed pattern as nonlinear within this cohort, but we do not interpret the apparent upper-range flattening as physiologic.

While these findings were unexpected and differed from the original hypothesis, the broader literature is mixed rather than uniformly contradictory. Palmer et al. (2007) reported no statistically significant association between high BMI and estrogen receptor-negative breast cancer among Black women [14]. At the same time, more recent epidemiologic and mechanistic studies support links between adiposity, aromatase-mediated estrogen production, lower SHBG, insulin resistance, systemic inflammation, and postmenopausal breast cancer risk [7-9,15-17]. Wang et al. (2022) found no association between anthropometric measures, including BMI, and breast cancer risk in postmenopausal women [18], whereas other recent cohort and meta-analytic work supports a positive association, particularly for estrogen receptor-positive postmenopausal disease [10,11]. Hormonal mechanisms may also contribute to age-related variation in breast cancer incidence, because many breast cancers are hormone-responsive and ovarian estrogen production declines after menopause. However, among postmenopausal women, adipose tissue remains an important source of estrogen through aromatase activity, particularly in women with obesity [8]. Therefore, declining ovarian estrogen with age may contribute to changes in incidence at older ages, but it does not by itself explain a flat BMI gradient in a cohort of women aged 55 years and older. Contemporary population data also show that breast cancer incidence increases with age until the seventh decade and then decreases, a pattern thought to partly reflect lower screening at older ages rather than ovarian physiology alone [19]. In addition, the population-based Iowa Women’s Health Study does not suggest that risk necessarily plateaus in the oldest women; among women aged 75 years or older, those in the highest BMI quartile had higher breast cancer risk than those in the lowest quartile [20]. The public-use data also did not include ovarian function, age at menopause, circulating estrogen, hormone therapy, or breast cancer receptor subtype. Thus, the present findings do not overturn the broader literature; the observed upper-range flattening is best interpreted cautiously as cohort-specific nonlinear attenuation, potentially influenced by selection, competing risk, or differential case capture, rather than evidence of a general biologic plateau.

These data also could not distinguish the breast cancer subtype. Although contemporary US data show lower overall breast cancer incidence but higher breast cancer mortality in Black women than in White women [19,21], triple-negative breast cancer (TNBC), which is less common but more aggressive, occurs more often in Black women [22,23], and Black women with TNBC may experience worse mortality than White women with TNBC [24,25]. Some exploratory studies have not shown a clear association between BMI and subtype-specific incidence in older women [18], whereas Choi et al. reported worse progression among overweight or obese women with TNBC after surgical resection [26]. Therefore, the lower overall incidence observed in the present study should not be interpreted as lower severity after diagnosis.

The present findings may also reflect the challenges of using clinical trial data as an observational cohort for etiologic questions. VITAL had strong overall follow-up in the parent trial [13], but differential case loss or under-ascertainment could still attenuate observed gradients if heavier women, older women, or Black women were less likely to undergo screening, diagnostic work-up, or complete follow-up. Women with obesity may face weight-related barriers to cancer screening [27], older women with cognitive impairment may be less likely to undergo mammography [28], and Black women may experience longer time to follow-up after abnormal mammography and greater attrition across the screening continuum [29,30]. These possibilities are consistent with interpreting the upper-range flattening as cohort-specific and methodologic rather than biologic.

Strengths and limitations

Strengths of this study include its relatively large cohort, prospective follow-up, and adjudicated breast cancer outcomes. However, as a secondary observational analysis of a selected clinical trial cohort, it was limited by the relatively small number of incident breast cancer cases, self-reported baseline height and weight, BMI measured only at baseline, and the absence of several potentially relevant covariates in the public-use data. The public-use dataset also did not include tumor subtype or receptor-status information, including estrogen receptor, progesterone receptor, HER2, or TNBC status, which limits etiologic interpretation and prevents assessment of whether the observed BMI and race patterns differed by biologically relevant breast cancer subtype. Residual confounding and differential selection, screening, follow-up, or case ascertainment by age, BMI, or race should therefore be considered when interpreting the observed nonlinear pattern.

## Conclusions

In this secondary analysis of the VITAL cohort, breast cancer incidence increased across lower BMI and age ranges but flattened at the highest BMI levels and the oldest ages. Taken together with the broader literature, this upper-range attenuation is better interpreted as a nonlinear pattern within this selected trial cohort than as evidence of a biologic plateau. More broadly, the findings illustrate how secondary observational analyses of clinical trial data may attenuate or distort expected exposure-disease gradients through selection, ascertainment, or competing-risk processes. These results, therefore, should not be taken as revising current knowledge regarding adiposity and breast cancer risk or as a basis for changing current prevention or screening recommendations.
